# Disease-associated microglia in neurodegenerative diseases: Friend or foe?

**DOI:** 10.1371/journal.pbio.3003426

**Published:** 2025-10-21

**Authors:** Yi-Hsuan Cheng, Margaret S. Ho

**Affiliations:** 1 Institute of Neuroscience, National Yang Ming Chiao Tung University, Taipei, Taiwan; 2 Brain Research Center, National Yang Ming Chiao Tung University, Taipei, Taiwan

## Abstract

Recent advances in single-cell transcriptomics have led to the identification of disease-associated microglia (DAM) as a distinct, conserved microglia state associated with mouse models of Alzheimer’s disease (AD) and amyotrophic lateral sclerosis, and with aging. DAM are characterized by downregulation of homeostatic genes and upregulation of lipid metabolism and phagocytosis genes, including key risk factors for AD in humans. Although characterized in models of AD, whether DAM acts as universal sensor across all neurodegenerative diseases remains unknown. This Essay discusses the dynamics, origins, and therapeutic potential of DAM in neurodegeneration, alongside evidence supporting a protective role for them in regulating disease processes.

## Abstract 2

Disease-associated microglia are a distinct, conserved microglial state associated with Alzheimer’s disease, amyotrophic lateral sclerosis and aging. This Essay discusses the dynamics, origins and therapeutic potential of these microglia in neurodegeneration, alongside evidence supporting a protective role for them in regulating disease processes.

## Introduction

Advances in research methodologies have profoundly transformed the way scientists perceive and study cells, shifting from population-level analyses to high-resolution, single-cell approaches that capture intricate details of individual cell states and their activation/deactivation patterns. In particular, single-cell RNA sequencing (scRNA-seq) has revolutionized our understanding of the cellular diversity in the brain, generating comprehensive atlases of brain cell types: insights that are unattainable through bulk RNA analyses. Single-cell technologies have provided critical information about how specific cell populations contribute to the onset and progression of neurodegenerative diseases, a group of disorders characterized by progressive neuronal loss, accumulation of misfolded protein aggregates, and a broad spectrum of motor and cognitive impairments [1–3]. While these advances have significantly deepened our understanding of the disease mechanisms, the development of effective therapeutic strategies remains an ongoing and formidable challenge.

In addition to the traditionally neuron-centric view on the mechanisms of neurodegenerative diseases, microglia (the resident immune cells of the brain) have emerged as key contributors to these diseases, making them promising targets for therapeutic intervention. Microglia arise from erythromyeloid progenitor cells that migrate from the yolk sac to the central nervous system (CNS), along with perivascular, meningeal, and choroid plexus macrophages that reside at the interface of the periphery and CNS [4,5]. As early as 1904 and 1910, Nissl and Alzheimer described the presence of amoeboid glial cells associated with certain pathological conditions, an observation confirmed in 1919 by Pío del Río Hortega using silver carbonate staining to identify these amoeboid cells as microglia in brain specimens from patients with conditions later known as Alzheimer’s disease (AD) [6–8]. These observations were the first to implicate a change in microglial morphology, likely mirroring shifts in their functional states, in neurodegenerative diseases.

Acting as a double-edged sword in the brain, microglia engulf neuronal debris and pathological aggregates, facilitating the clearance of harmful materials. Beyond this role, microglia regulate neuroinflammation by releasing various cytokines, including pro-inflammatory cytokines such as IL-1β and TNF, as well as anti-inflammatory mediators such as IL-4 and arginase-1 (Arg1). Based on the nature of cytokine release, a classical dichotomy of microglial activation—“M1” (pro-inflammatory and neurotoxic) versus “M2” (anti-inflammatory and neuroprotective)—was proposed [9]. However, this binary classification is now considered an oversimplification in light of growing evidence supporting a highly heterogeneous and dynamic microglial landscape. Several distinct types of microglia have been described. Bipolar/rod-shaped microglia, first reported by Franz Nissl in 1899, represent a transitional morphology characterized by high levels of proliferation and phagocytic activity; functions that may confer neuroprotective effects [10–12]. Other reported microglia subtypes include dystrophic (senescent) [13] and dark microglia [14], which have been identified by their distinct transcriptional profiles and by their ultra-structures, respectively.

Building on these morphological observations, genome-wide expression profiling has revealed further complexity in microglial states. Rather than adhering to “M1” or “M2” phenotypes, microglia exist along a dynamic continuum, often displaying mixed or intermediate phenotypes with unique expression profiles of genetic markers. This plasticity reflects the context-dependent nature of microglial responses [15–17]. Notably, the identification of disease-associated microglia (DAM) has underscored the existence of specialized microglial states with distinct transcriptional signatures tailored to specific disease conditions. In this Essay, we outline the discoveries that led to the identification of DAM, highlighting their defining features, functional significance, and disease relevance. We also examine key discrepancies and unresolved questions, and advocate for a predominantly neuroprotective role of DAM.

## Identification and characterization of DAM

The advent of single-cell transcriptomics revolutionized the study of neurodegenerative diseases, enabling researchers to dissect cellular heterogeneity in diseased brains with unprecedented resolution. Around 2017, several studies reported distinct microglial populations with unique transcriptional profiles identified by scRNA-seq analyses of sorted immune cells (typically CD45⁺ or CD11b⁺) from control and diseased brains, in mouse models of AD and amyotrophic lateral sclerosis (ALS), and in aging [18–20]. Two distinct microglial clusters were identified in 5xFAD mice (Box 1) [21]. These DAM display downregulated expression of core homeostatic genes [22–26] and upregulated expression of genes involved in lipid metabolism and phagocytosis [27–30] (Table 1). Among these upregulated genes are several known AD risk factors, including *Apoe* [31,32], *Ctsd* [33], *Lpl*, *Trem2* [34], and *Tyrobp*, which encodes the TREM2 adaptor protein [35]. The microglial populations in these clusters expand progressively towards the later stages of disease in mouse models of AD (5xFAD) and ALS (SOD1^G93A^; Box 1) [36], and in aging [19]; they are also detected in other disease models, including AD tauopathy [18,37,38].

**Table 1:** Characteristics of disease-associated microglia in mice.

Microglial subtype	Characteristic gene markers	Key features
DAM	Upregulated: *Apoe, Lpl, Cst7, Ctsd, Trem2, Tyrobp*; downregulated: *P2ry12/P2ry13, Cx3cr1, Tmem119.*	Enhanced phagocytosis and lipid metabolism.
Pro-inflammatory DAM	Upregulated: *Cd44, Il1b, Nfkb, Stat1, Tlr2.*	Correlate with neuropathology; appear in the early disease stage.
Anti-inflammatory DAM	Upregulated: *Apoe, Atf1, Cxcr4, Igf1, Lxra/b.*	Appear in the later disease stage.
MgnD microglia	Upregulated: *Apoe, mir-155, Clec7a*; downregulated: *Tgfb, P2ry12, Tmem119* and homeostatic genes.	Transcriptionally similar to DAM yet distinct from the homeostatic (M0) and pro-inflammatory (M1) microglia.
PAM	Upregulated: *Spp1*, *Gpnmb*, *Igf1*, *Clec7a*, *Lpl*, *Cd9*, *Cd63*, *Lgals3*, *Fabp5*, *Itgax*, *Apoe, Tyrobp*; downregulated: homeostatic genes	Early postnatal microglia sharing similar transcriptional signatures with DAM, found in the developing white matter.
Sensome-expressing microglia	Enriched with *P2ry12, Tmem119, Gpr34, Csf1r, Cx3cr1, Dap12.*	Exhibit increased expression of genes involved in microbial recognition, host defense, and neuroprotection.
Primed microglia	Upregulated: *Apoe, Axl, Clec7a, Itgax* (also known as *CD11c*), *Lgals3* (also known as *Galectin-3* and *Mac2*); downregulated: homeostatic genes.	Emerges in response to aging and neurodegeneration and differs transcriptionally from “M1” microglia despite their secretion of pro-inflammatory cytokines, chemokines, and reactive oxygen species.
HAM	Upregulated: *APOE*, *ABCA7*, *GPR141*, *PTK2B*, *SPI1*, *ZYX*; downregulated: *MEF2C.*	Bear little resemblance to DAM.
Mic1	Enriched with AD risk genes and the MHC-II genes *CD74* and *HLA-DRB1.*	Share similar transcriptional signatures with DAM but not with aged microglia.

AD, Alzheimer’s disease; DAM, disease-associated microglia; HAM, human Alzheimer’s microglia; MgnD, microglial neurodegenerative phenotype; Mic1, human AD-associated microglia subpopulation; PAM, proliferation-region-associated microglia.

**Box 1. Mouse models of neurodegenerative diseases.**

The genetic nmes and descriptions of mouse models of different neurodegenerative diseases are listed below.

**5xFAD**

A model of Alzheimer’s disease (AD) in which mice express five familial AD mutations in the human amyloid precursor protein (APP) and presenilin-1 (PS1) that models β-amyloid (Aβ) pathology.

**APP–PS1**

A model of AD in which mice overexpress mutated genes for APP and PS1.

**TauPS2APP**

A triple-transgenic mouse model used to study AD, simultaneously expressing mutant human forms of APP, Presenilin 2 (PSEN2), and human Tau (h-tau).

**PS19 tauopathy**

A model of AD tauopathy that incorporates a single mutant human MAPT transgene under the control of the PrnP promoter.

**SOD1** ^**G93A**^

A model of amyotrophic lateral sclerosis (ALS) in which mice express a human Cu-Zn superoxide dismutase mutation at glycine 93 to alanine.

**rNLS8**

A model of ALS in which mice express a human TDP-43 transgene that lacks a proper nuclear localization signal (hTDP-43ΔNLS).

**EAE**

Experimental autoimmune encephalomyelitis, a mouse model of multiple sclerosis (MS).

**Cuprizone model of MS**

A non-autoimmune mouse model of MS induced by feeding mice the copper-chelating toxin, cuprizone.

Thus, DAM represents a group of microglia characterized by enhanced phagocytic activity and lipid metabolism that is transformed into a disease-associated state upon losing their homeostatic features. Notably, despite variations in sequencing depth, immune cell isolation strategies, and genetic backgrounds used to model disease, DAM have been consistently identified. This consistent appearance across different disease models (including β-amyloid [Aβ]-driven AD, tau-driven AD, and ALS) suggests that DAM might represent a generalizable, pathology-responsive microglial state with potential implications for both disease progression and therapeutic intervention. Nevertheless, whether DAM observed in different disease models or in aging are identical cell populations remains largely unexplored.

## Heterogeneity in DAM and DAM-like microglia

Weighted gene co-expression network analysis applied to existing microglial gene expression datasets has been used to predict distinct pro-inflammatory and anti-inflammatory subsets within DAM (Table 1), highlighting their heterogeneity [39,40]. Pro-inflammatory DAM emerge at an early stage of disease and positively correlate with neuropathology; by contrast, anti-inflammatory DAM become more prominent at later stages of disease. Despite these DAM subtypes being identified, it remains unclear whether microglia transit between these states. The results of one study that used trajectory analyses have suggested that homeostatic microglia differentiate into different states, but whether microglia interconvert between these states remains to be fully elucidated [41].

Complementing this view, scRNA-seq analyses have identified multiple microglial clusters that differentially express DAM-associated genes, including *Apoe*, *Lpl*, *Cd9*, *Cst7*, and *Trem2*, suggesting the existence of intermediate states between homeostatic microglia and fully activated DAM. A continuum of transcriptional states has been shown among *Itgax* (CD11c)-positive microglia isolated from 5xFAD mice at different stages of disease progression, identifying a transitional population that bridges the homeostatic and DAM phenotypes [19]. In this intermediate state, a subset of DAM genes is modestly upregulated (excluding most genes involved in lipid metabolism and phagocytosis), while homeostatic genes are concurrently downregulated. These observations support a model in which DAM represents a mature activation state that evolves through a progressive transcriptional shift originating in homeostatic microglia.

In parallel, a separate study using similar transcriptomic approaches identified distinct microglial clusters associated with neurodegenerative conditions, termed the microglial neurodegenerative phenotype (MgnD) [20]. These clusters emerge during aging and in mouse models of ALS (SOD1^G93A^), AD (APP–PS1; Box 1), and multiple sclerosis (MS; Experimental autoimmune encephalomyelitis [EAE]). MgnD microglia share a similar transcriptional profile with DAM (Table 1), including upregulation of *mir-155*, a downstream target of *Apoe*. Supporting the transcriptomic results, local injection of apoptotic neurons into the cortex and hippocampus of control mice recruits MgnD-like microglia: upon phagocytosis of apoptotic cells, P2ry12-positive homeostatic microglia transform into a phagocytic MgnD-like state, marked by increased expression of *Apoe* and reduced expression of homeostatic genes. Importantly, MgnD microglia are distinct from the classic pro-inflammatory “M1” microglia, which express *Egr1* but lack *Apoe*. These findings highlight MgnD as a unique microglial subset; they are transcriptionally similar to DAM yet distinct from the homeostatic (M0) and pro-inflammatory (M1) microglia.

Intriguingly, a subset of early postnatal microglia found in the developing white matter, named proliferation-region-associated microglia (PAM), also shares similar transcriptional signatures with DAM (Table 1) [42]. PAM exhibit amoeboid morphology, are metabolically active, and phagocytose newly formed oligodendrocytes. The parallels between DAM and PAM support the concept that genes expressed during development are reactivated in aging and degeneration [43]. Furthermore, aging itself profoundly reshapes the microglial transcriptome, giving rise to sensome-expressing microglia (Table 1) that share transcriptional features with DAM and exhibit increased expression of genes involved in microbial recognition, host defense, and neuroprotection [24]. Enriched with several aging-associated genes, DAM also display features of cellular senescence and may arise as a consequence of early microglial proliferation [19,20,44–46]. To this end, a distinct microglial population, termed primed microglia (Table 1), emerges in response to aging and neurodegeneration and differs transcriptionally from “M1” microglia despite their secretion of pro-inflammatory cytokines, chemokines, and reactive oxygen species. While the exact relationship between primed microglia and DAM remains unclear, their overlapping gene expression profiles suggest shared phagocytic and immune-responsive features shaped by age and neurodegeneration-related pathology [47–49].

In our view, these distinct microglial populations—although sharing certain transcriptional features—likely represent different functional states of microglia. They are identified in distinct cellular contexts, suggesting functions specific to each. For example, sensome-expressing and primed microglia are associated with aging, yet whether they actively combat age-related changes or merely arise as a consequence of the aging program remains unclear. While some genes are commonly expressed across these populations, such overlaps may reflect shared cellular programs for initiating or sustaining these states rather than indicating a common function. Importantly, DAM have so far been defined exclusively by scRNA-seq transcriptional profiles, with no functional studies yet clarifying their states. Given that scRNA-seq exhibits low sensitivity for detecting DAM genes in human postmortem brains [50], complementary approaches such as spatial transcriptomics or direct manipulation using inducible genetic tools are needed to fully characterize DAM.

## Trem2–ApoE signaling orchestrates DAM activation

**Fig 1:**
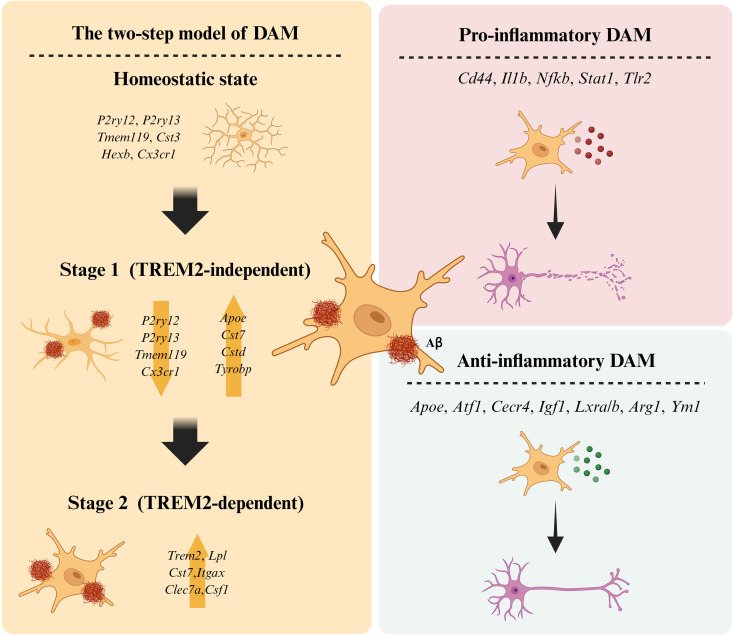
A two-step transition for Disease-associated microglia (DAM) activation. DAM are proposed to transform from homeostatic microglia via a two-step program. In the stage 1 intermediate state, expression of many homeostatic genes is downregulated, and the expression of a subset of DAM-associated genes, including *Apoe*, begins to increase. For DAM to be fully activated, a Trem2-dependent signal commences and transforms the stage 1 intermediate state to stage 2 DAM, which acquires full phagocytic capacity. Alternatively, DAMs can be grouped into subsets (pro- and anti-inflammatory DAM) according to their inflammatory properties. Figure created with BioRender; https://BioRender.com.

Emerging evidence implicates the Trem2–ApoE signaling axis as a key regulator of DAM activation. Genetic variants in both *TREM2* and *APOE* are linked to increased AD risk, and their expression is elevated in murine DAM [19,20,27,32,34,51]. Intriguingly, the intermediate DAM cluster is expanded in *Trem2*-deficient mice, indicating that Trem2 is required for full DAM activation. While *Apoe* expression is still induced in the *Trem2*-deficient intermediate microglia by an unknown mechanism, a two-step transition is hypothesized to orchestrate DAM activation: an initial Trem2*-*independent signal that induces *Apoe* expression and suppresses homeostatic gene expression, leading to partial activation of the DAM program (stage 1 intermediate state). Consequently, a Trem2-dependent signal further activates the intermediate DAM program to achieve its full capacity by acquiring phagocytic and lipid metabolism activity (stage 2 full activation) (Fig 1) [52].

Studies on the Trem2–ApoE signaling axis have sparked debate over its role in DAM activation. As Trem2–ApoE signaling regulates various aspects of microglial development, function, and survival, it remains unclear whether this pathway uniquely drives DAM activation or acts through indirect mechanisms such as lipid or glucose metabolism [53]. Furthermore, it is still unresolved whether Trem2–ApoE signaling exerts predominantly neuroprotective or neurotoxic effects in the context of neurodegeneration. For instance, deficiency of Trem2 or ApoE appears to mitigate neuron loss in a mouse model of axotomy-induced facial motor neuron injury [20]. While levels of *Apoe* expression correlates with neuronal loss [54], the protein also accumulates in amyloid plaques, stimulates amyloid precursor protein (APP) transcription, and facilitates Aβ aggregation in AD mouse models [20,55,56]. *App* and *Apoe* expression are also upregulated in microglia from mice with EAE and mouse models of ALS [57]. Impaired Trem2 signaling, either via the AD-associated *Trem2* variant or genetic deletion, also results in reduced brain atrophy and microgliosis in mice with PS19 tauopathy (Box 1) [37,38], further supporting the view that this pathway may exacerbate disease processes.

By contrast, some studies suggest that Trem2–ApoE signaling is neuroprotective in the context of DAM activation. Loss of ApoE de-represses homeostatic gene expression in microglia while simultaneously blocking the induction of DAM-associated genes in MgnD-like microglia [20]. In addition, the majority of loss-of-function studies indicate that Trem2 deletion exacerbates disease progression by suppressing DAM-associated gene expression [19,20,58] and promoting a shift toward a pro-inflammatory “M1” microglial state [59]. Thus, reduced DAM polarization in *Apoe*- or *Trem2*-deficient microglia might cause microglia to assume potentially harmful states, suggesting that Trem2–ApoE signaling positively regulates and promotes protective DAM functions in disease contexts.

## DAM–plaque association and pathological implications

A prominent feature of DAM is their preferential localization near protein aggregates, such as Aβ plaques in AD. Using immunohistochemistry and single-molecule fluorescence in situ hybridization, DAM have been identified clustering around Aβ plaques in the cortex of 5xFAD mice [19]. Similar DAM–plaque associations have been observed in other mouse models of neurodegeneration and during aging [19,20,60]. The close proximity of DAM to Aβ plaques may serve a dual purpose: physically limiting the spread of plaques to protect surrounding neurons from Aβ-induced neurotoxicity and functionally contributing to their clearance by the enhanced phagocytic activity of DAM.

Closely linked to its role in DAM activation, Trem2 is essential for proper microglial clustering around Aβ plaques. In *Trem2*-deficient mouse models of AD, DAM fail to localize to plaques [19,59]. Particularly, Trem2 deletion in TauPS2APP mice (Box 1)—which drives Aβ production and enhances tau phosphorylation—alters plaque-associated pathology, including reduced plaque-associated and overall microgliosis, increased X-34-positive plaque diffuseness, and reduced Aβ plaque accumulation at later stages of disease [59,61]. Additionally, levels of circulating soluble toxic Aβ are increased, suggesting enhanced diffusion [59]. Moreover, plaque-associated ApoE senses IL-33-induced VCAM1, positively directing microglial chemotaxis to the plaques [62]. Altogether, these findings highlight Trem2–ApoE signaling as a critical regulator of DAM–Aβ plaque interactions: microglial Trem2 activity seems to limit Aβ-induced tau phosphorylation, aggregation, and spreading, as reflected by preserved plaque integrity [59,63–66].

Strikingly, the failure of DAM to associate with plaques results in pronounced pathological outcomes, including greater plaque-associated and total axonal dystrophy, along with reduced brain volume [59,67]. Considering that Trem2 deletion in mouse models of AD suppresses DAM gene expression, disrupts microglial clustering around Aβ plaques, and exacerbates plaque diffusion and amyloid burden [59,65,66,68–70], it is plausible to speculate that these neurodegenerative effects stem from Trem2-mediated DAM dysregulation, even in light of the complex and multifaceted roles of Trem2 [19,65–67,71,72]. We argue that the phenomenon of DAM–plaque association should be given greater weight in defining DAM, moving beyond definitions based on single-cell transcriptomics. Thus far, immunostaining approaches have been used to define the spatial association between DAM and plaques, but these methods can be limited by marker selection and resolution. Spatial transcriptomics, by preserving spatial context, offers the means to verify or refine such associations and thereby strengthen the criteria for DAM identification. Importantly, it can also uncover transcriptional heterogeneity within plaque-associated DAM in a spatial manner, revealing potential subpopulations with distinct functional roles in disease progression.

## DAM in other neurodegenerative diseases

Although DAM were initially characterized in mouse models of AD, their presence has also been observed in other neurodegenerative diseases, including ALS, MS, and Parkinson’s disease (PD), both in animal models (Box 1) and human tissues [19,57,73–78]. Transcriptomic analyses of purified microglia or immune cells from SOD1^G93A^ mice (a model of ALS) reveal gene expression changes overlapping with those observed in mouse models of AD, including an age-associated DAM expansion [19,57]. Likewise, DAM-like microglia with similar transcriptional characterization have been detected in the EAE and cuprizone models of MS [75–78]. In the rNLS8 model of ALS, in which human TDP-43 (hTDP-43) pathology can be reversibly induced in neurons, suppression of hTDP-43 triggers the proliferation of DAM. These microglia selectively clear neuronal hTDP-43 aggregates, suggesting a neuroprotective role [73]. In the absence of *Trem2*, hTDP-43 accumulates due to the impaired microglial clearance and a failure to activate DAM [74].

Early evidence for DAM presence in PD came from the observation of robust MHC-II staining in the substantia nigra of brains of patients with PD, indicating that microglia are reactive [79]. With the advent of scRNA-seq, DAM in PD has been more definitively characterized in mouse models. For instance, scRNA-seq analyses of purified Cx3cr1-positive cells from mice expressing human α-synuclein—a key aggregate component in PD—reveal an expanded microglial population expressing DAM-associated genes such as *Cst7*, *Lpl,* and *Apoe* [80]. Similarly, other studies have reported DAM-like transcriptional profiles in α-synuclein-expressing microglia, marked by increased expression of *Apoe* and pro-inflammatory genes [81]. An increase in the number of substantia nigra microglia exhibiting an amoeboid, reactive morphology has also been identified in postmortem brain tissue from patients with PD [82]. These microglia express genes such as *GPNMB*, further aligning them with the DAM phenotype. Intriguingly, *Trem2* deficiency aggravates α-synuclein-induced neurodegeneration and neuroinflammation in mouse models of PD, and the rare p.R47H variant has been associated with frontotemporal dementia and PD [83,84]. Together, these findings support the existence of a *Trem2*-dependent DAM program in PD, implicating shared microglial reactive states across neurodegenerative diseases.

Nevertheless, caution should be taken when defining DAM in neurodegenerative diseases other than AD. Although comparable transcriptional signatures are observed, microglial populations in different disease contexts respond to distinct pathological challenges and may therefore diverge functionally. Key uncertainties remain regarding the signaling pathways that initiate or sustain their activation, and whether they undergo intermediate or full transitions into DAM. Without clarifying these mechanisms, there is a risk of oversimplifying DAM biology and overlooking disease-specific microglial responses. These considerations point to both shared and disease-specific aspects of DAM, underscoring the need for refined definitions across conditions.

## DAM in diseased human brains

Although extensive efforts have been made to elucidate the pathophysiology of neurodegenerative diseases using various animal models, their translational relevance to human pathology remains variable [76]. Comparative scRNA-seq analyses of CD11b^-^, CD45^-^ or Cx3cr1::GFP-positive cells from diseased tissues of mice and humans consistently demonstrate an expansion of DAM gene modules in both species, supporting the translational validity of murine DAM signatures (Table 1) [18]. Complementary evidence from cortical brain tissues of patients with AD further reveals that DAM express *LPL* and harbor thioflavin-S-positive Aβ deposits, indicative of active phagocytosis, reinforcing functional conservation across species [19]. Moreover, scRNA-seq comparisons of healthy, AD, and PD human striata have identified microglial subsets with shared molecular signatures with murine DAM that are unique to each disorder, with regional differences [85]. Consistent with this, regional and age-related transcriptomic differences in microglia from patients with AD have been reported, particularly involving a wide range of pro-inflammatory pathways [86]. These findings suggest that while microglial responses are functionally conserved across species, they are modulated in a region- and disease-specific manner in the human brain. Furthermore, they support our view that DAM or other microglial populations under different terminologies are intrinsically different, despite the fact that they share some similar transcriptional features.

By contrast, some reports have shown limited overlap between differentially expressed genes in brain tissue from patients with AD and those from mouse DAM or MgnD, with relatively few DAM genes detected in human datasets and certain AD-associated genes appearing to be human-specific [72,87,88]. In fact, these studies found no significant downregulation of homeostatic microglial genes or upregulation of DAM genes in tissues from patients with AD [72,88]. In addition, one study reported DAM gene upregulation only in mouse models of AD and ALS, but not in human precuneus samples, even though homeostatic gene expression was consistently downregulated in both species [54]. Notably, human-specific microglial subtypes have been identified. These include human Alzheimer’s microglia, characterized by transcriptomic features associated with both aging and AD [89], and Mic1, a unique subpopulation enriched for AD risk genes such as *APOE* and *TREM2*, which seems to be distinct from microglia in normal aging [87]. It remains unclear whether these human-specific populations are analogous to murine DAM or represent fundamentally different states. These inconsistencies may stem from the use of postmortem, end-stage human tissues that fails to accurately capture disease dynamics. Moreover, anatomical heterogeneity between the human and mouse brain samples complicates direct comparisons.

Human genetic studies have linked hypomorphic *TREM2* variants to an increased risk of AD, spurring investigations into its role in DAM within the human brain [34,51,90]. Consistent with observations in *Trem2*-deficient mice, patients with AD carrying rare TREM2 variants (such as R47H or R62H) exhibit impaired microglial clustering around Aβ plaques. This deficiency is associated with an increase in dystrophic neurites, exacerbation of tau pathology, and enhanced seeding and spreading of neuritic plaque-associated tau aggregates [20,66,68,91,92]. These findings underscore a conserved and protective role for TREM2 in both murine and human neurodegenerative brains: TREM2 facilitates DAM activation, promotes microglial clustering around plaques, and contributes to the formation of a neuroprotective barrier that limits Aβ plaque expansion and reduces plaque-associated neurotoxicity.

## A neuroprotective function for DAM?

**Fig 2:**
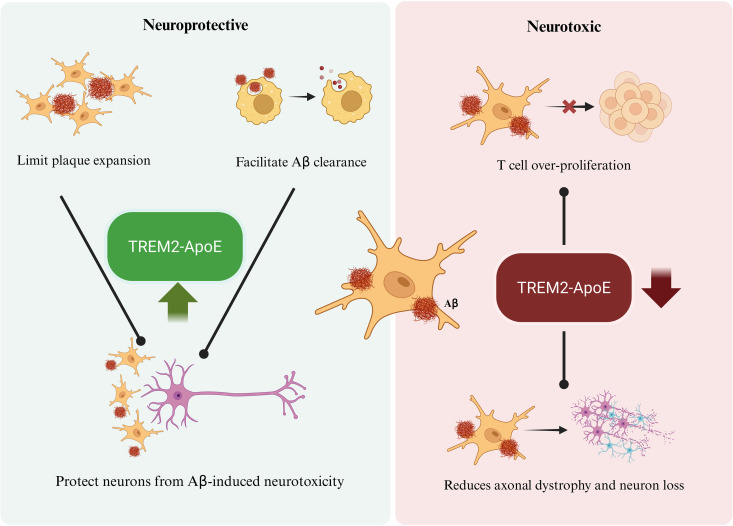
Neuroprotective and neurotoxic functions of disease-associated microglia (DAM). DAM are considered neuroprotective because they function as a physical barrier to limit plaque diffusion and because they phagocytically clear plaque-associated aggregates (β-amyloid or hTDP43 aggregates). Both mechanisms would lead to the protection of surrounding neurons from plaque-related neurotoxicity. A few studies have also pointed to a neurotoxic role for DAM. In these contexts, the presence of DAM causes increased T cell proliferation and pro-inflammatory signals in the brain, and DAM deactivation in the absence of Trem2–ApoE signaling reduces neuronal loss and axonal dystrophy. Figure created with BioRender; https://BioRender.com.

Multiple lines of evidence support the notion that DAM are neuroprotective in mouse models of AD. Murine DAM express classic “M2”-like anti-inflammatory markers such as Arg1 and chitinase-3-like protein 1 (Ym1), alongside ApoE [20]. Expression of the secreted neurotrophic factor insulin-like growth factor 1 is also upregulated in DAM [93–95]. Notably, changes in DAM gene expression show no direct correlation with the extent of neuronal loss, suggesting that DAM do not promote neuronal cell death [20,54,96]. In line with these findings, DAM accumulate around Aβ plaques and help contain the spread of tau pathology, reinforcing a neuroprotective role for DAM (Fig 2).

Interestingly, the results of early studies also support the neuroprotective interpretation. For example, Ym1-positive “M2” microglia cluster around Aβ plaques at 6 months of age in the hippocampus of APP–PS1 mice. These microglia have high phagocytic activity and low expression of pro-inflammatory genes [97]. By 18 months of age, “M1”-like microglia become predominant, coinciding with increased neurodegeneration and a decline in phagocytic activity [98]. This “M2”-to-“M1” shift suggests that the early, plaque-associated “M2”-like microglia—likely precursors or equivalents of DAM—have a critical protective role that diminishes over time as disease progresses.

Given the essential role of Trem2 in DAM induction and function, agonistic Trem2 antibodies have been evaluated in mouse models of Aβ pathology. These treatments enhance microglial proliferation and reduce plaque-associated dystrophic neurites, highlighting the therapeutic potential of Trem2-mediated DAM activation [99–101]. By contrast, blocking early microglial proliferation can prevent DAM activation while simultaneously reducing Aβ pathology [46]. This paradoxical outcome suggests that DAM may also contribute to disease progression under certain conditions. As mentioned above, heterogeneity exists within the DAM population, with subpopulations differing in inflammatory profiles and phagocytic capacities. These subsets may coexist in both temporal and spatial dimensions and contribute to either beneficial or detrimental outcomes depending on the microenvironment [39,40]. Furthermore, DAM-like microglia resembling the MgnD phenotype fail to suppress T cell proliferation and instead amplify pro-inflammatory T cell responses [20]. These findings underscore the context-dependent nature of DAM activation and function, highlighting the need for therapeutic strategies that selectively harness beneficial DAM responses while limiting their potential pathogenic effects.

Although concerns have been raised regarding whether chronic DAM activity might contribute to neuroinflammation, such speculation should not obscure their fundamental role as pathology responders. The recurrent enrichment of genes regulating phagocytosis, lipid metabolism, and lysosomal activity underscores a purposeful transcriptional reprogramming toward debris clearance. Their preferential clustering around amyloid plaques or dystrophic neurons represents a coordinated effort to contain injury rather than to promote it. On the basis of these considerations, we argue that DAM should therefore not be pathologized, nor regarded as drivers of disease; rather, they should be recognized as a central line of defense—adaptive, functional, and potentially therapeutic if properly supported.

## Conclusions and future perspectives

The discovery of DAM and related microglial subsets has fundamentally reshaped our understanding of microglial responses in neurodegenerative diseases. Single-cell transcriptomic profiling reveals that DAM—characterized by downregulation of homeostatic genes and upregulation of genes involved in lipid metabolism and phagocytosis—are key players emerging across various mouse models of disease, including Aβ-driven and tau-driven AD, and ALS, as well as models of aging. Additional microglial subsets with transcriptional overlap to DAM have been identified under distinct pathological contexts, underscoring the complexity of microglial states beyond the classic “M1”/“M2” framework. Although DAM and DAM-like microglial subsets might differ in functional responses in a context-dependent manner, these findings position DAM as conserved, pathology-responsive sensors across neurodegenerative conditions, reinforcing their potential as therapeutic targets.

Rather than a uniform entity, DAM likely represents a spectrum of states shaped by disease context, progression, environment, and genetics. Yet, critical questions remain regarding the triggers and timing of DAM activation, the existence of intermediate states, their regional specificity, and their interactions with plaques, neurons, and other glia (Box 2). Resolving these issues will require longitudinal single-cell profiling, fate mapping, and spatial transcriptomics, together with in vivo perturbations to define whether DAM are protective, pathogenic, or context-dependent.

**Box 2. Unknown aspects of DAM biology.**

**DAM: Drivers or responders?**

One of the most pressing questions is whether disease-associated microglia (DAM) actively drive pathogenesis or instead reflect a reactive state to neuronal injury. Disentangling causality will require temporally resolved approaches, including inducible, cell-type-specific genetic models and in vivo imaging to track DAM dynamics in real time. For instance, the recently developed inducible *Cst7*^*DD-Cre*^ mice provide a promising tool for directly manipulating DAM function [102], offering a path to determine whether these cells are pathogenic, protective, or context-dependent.

**Functional heterogeneity and plasticity**

Current data suggest that DAM are not a uniform population. Sub-clustering analyses and gene co-expression networks have revealed subsets with potentially pro- or anti-inflammatory functions. However, whether these subsets represent stable, lineage-specific populations or transient states along a dynamic continuum is unknown. Furthermore, whether DAM revert to homeostatic microglia or are locked in a disease state is also uncertain. Fate-mapping and single-cell lineage tracing will be critical in resolving questions about their origin and plasticity.

**Mechanisms of Trem2–ApoE signaling in DAM**

The Trem2–ApoE pathway has emerged as a key regulator of DAM induction, yet the precise mechanisms involved remain unclear. Given its multifaceted roles in microglial development, function, and survival, it is critical to determine how this pathway specifically drives the DAM program. Refined experimental models, including inducible or region-specific perturbations, will be essential to dissect these context-dependent effects and clarify the pathway’s unique role in DAM biology.

**The translational gap between mouse and human DAM**

Notable transcriptomic differences exist between murine and human microglia in neurodegenerative contexts. Certain DAM-associated genes identified in mouse models are either absent or regulated differently in postmortem human brains. This discrepancy may result from species-specific biology, end-stage sampling artifacts, or anatomical heterogeneity. Future studies using longitudinal human samples, induced pluripotent stem cell-derived microglia, and humanized mouse models will help bridge this translational gap.

**Crosstalk with other brain cell types**

Most DAM research has focused on microglia in isolation, yet neurodegeneration is inherently multicellular. DAM behavior is likely shaped by signals from reactive astrocytes, stressed neurons, and dysfunctional oligodendrocytes. These intercellular interactions remain poorly characterized in neurodegenerative contexts. Integrative multi-omics approaches such as scRNA-seq, spatial transcriptomics, and ligand-receptor mapping, in combination with other cell-type-specific tools, are needed to reveal the broader cellular network that modulates DAM identity and function.

**Therapeutic modulation: Help or harm?**

In addition to their heterogeneity, DAM seem to exert stage-dependent functions. Whereas microglia at an early stage of disease—possibly DAM associated with plaques—may promote clearance of pathological aggregates, subsets arising at later stages of disease exhibit pro-inflammatory features that can exacerbate chronic neuroinflammation. This temporal divergence underscores the need for therapeutic strategies that discriminate between protective and detrimental microglial states. Interventions targeting DAM, including Trem2 agonists, metabolic reprogramming, and transcriptional re-wiring, are currently under investigation, yet their efficacy, context specificity, and long-term safety remain unresolved.

Ultimately, delineating their trajectories, interactions, and functional boundaries will be key to harnessing DAM as precision targets for neurodegenerative disease therapy, with the promise of delaying—or potentially reversing—disease progression. Moving forward, integrating single-cell and spatial transcriptomics with functional assays in diverse disease models will be essential to resolve the heterogeneity of DAM and DAM-like microglial subsets. Moreover, understanding how systemic factors, aging, and intercellular crosstalk shape DAM activation will provide the mechanistic insights necessary to develop interventions that are both effective and context-specific.

## Abbreviations

Aβ: β-amyloid
AD: Alzheimer’s disease
ALS: amyotrophic lateral sclerosis
APP: amyloid precursor protein
Arg1: arginase-1
CNS: central nervous system
DAM: disease-associated microglia
EAE: experimental autoimmune encephalomyelitis
HAM: human Alzheimer’s microglia
MgnD: microglial neurodegenerative phenotype
MS: multiple sclerosis
PAM: proliferation-region-associated microglia
PD: Parkinson’s disease
scRNA-seq: single-cell RNA sequencing
